# Characterization of Clinical Isolates of *Bartonella henselae* Strains, South Korea

**DOI:** 10.3201/eid2405.171497

**Published:** 2018-05

**Authors:** Hea Yoon Kwon, Young Kyoung Park, Sun Myoung Lee, Ji Hyeon Baek, Jae-Seung Kang, Moon-Hyun Chung, Eun Ji Kim, Jin-Soo Lee

**Affiliations:** Inha University School of Medicine, Incheon, South Korea (H.Y. Kwon, Y.K. Park, S.M. Lee, J.H. Baek, J.-S. Kang, E.J. Kim, J.-S. Lee);; Jeju National University, Jeju, South Korea (M.-H. Chung)

**Keywords:** Bartonella henselae, Bartonella infection, cat-scratch disease, genetic analysis, bacteria, zoonoses, South Korea

## Abstract

*Bartonella henselae*, a gram-negative bacterium, is a common causative agent of zoonotic infections. We report 5 culture-proven cases of *B. henselae* infection in South Korea. By alignment of the 16S rRNA sequences and multilocus sequencing typing analysis, we identified all isolates as *B. henselae* Houston-1 strain, which belongs to sequence type 1.

The genus *Bartonella* includes infectious, gram-negative, facultative intracellular bacteria of numerous species. Among the *Bartonella* species, *B. henselae* is known as one of the most noteworthy pathogens (*1*). *B. henselae* causes cat-scratch disease, which is a common zoonosis and manifests various clinical symptoms (*2*).

A case of *B. henselae* infection in South Korea was confirmed in 2005 by PCR (*3*). Although a few more studies have been published after this case of *B. henselae*, only 2 cases were culture-proven: 1 from blood and 1 from bone marrow (*4*,*5*). Because of difficulties in cultivation and isolation, studies of the isolation of *B. henselae* from clinical specimens remain scarce. In this study, we analyzed the characteristics of the isolated *B. henselae* strains in South Korea and compared the clinical features of the patients.

## The Study

We conducted the study among patients who visited Inha University Hospital, a tertiary hospital in Incheon, South Korea, during 2009–2016. From these patients, we isolated 5 cases in which *B. henselae* was identified from cultures of blood or bone marrow (Table 1).

**Table 1:** Demographic and clinical characteristics of 5 case-patients whose serum sample cultures revealed the presence of *Bartonella henselae*, South Korea*

Characteristic	Case-patient 1	Case-patient 2	Case-patient 3	Case-patient 4 (*5*)	Case-patient 5 (*4*)
Age, y/sex	22/M	40/F	52/F	42/M	73/F
Clinical symptoms	Inguinal LAP, rash	Fever, myalgia	Febrile sense, left flank pain	Rash, fever, myalgia	Fever, general weakness
Lymphadenopathy	External iliac chain, inguinal area, supraclavicular area	Left neck level IV, V	Right neck II, III, VA	Right supraclavicular area	None
Leukocytes, cells/µL	10,920	6,130	8,180	19,260	5,120
AST/ALT, IU/dL	132/270	107/51	30/16	212/246	47/56
ESR,mm/h/CRP, mg/dL	21/3.93	44/5.5	4/0.14	25/12.9	22/13.16
Treatment	Third-generation cephalosporin, doxycycline	Levofloxacin, metronidazole, third-generation cephalosporin and doxycycline	Third-generation cephalosporin, minocycline, metronidazole	Doxycycline, changed to minocycline	Third-generation cephalosporin, doxycycline
*B. henselae* IgG titer	1:160	1:640	1:160	1:1,280	1:160
Pets	None	None	None	None	None
Co-occurring conditions	None	Pulmonary tuberculosis	None	None	None
*ALT, alanine aminotransferase; AST, aspartate aminotransferase; CRP, C-reactive protein; ESR, erythrocyte sedimentation rate.					

Case-patient 1 (IIBC1301) was a 22-year-old man hospitalized for left inguinal lymphadenopathy that had started 10 days earlier. His body temperature was 38.5°C, and he had rashes that started on the palms and soles and subsequently spread to his entire body. *B. henselae* was isolated from the blood that was cultured on the second day of hospitalization.

Case-patient 2 (IIBC1302) was a 40-year-old woman hospitalized for fever and myalgia, symptoms that had lasted for 1 month. The patient had an erythematous papular rash on her face and extremities and tenderness in her abdomen. Computed tomography (CT) of the abdomen showed chronic cholecystitis; therefore, levofloxacin and metronidazole were prescribed (Technical Appendix Figure, panel A). *B. henselae* was identified from cultures of blood obtained on the first day of the hospitalization. The patient had not raised any animals. After discharge, the patient experienced continuous fever, poor oral intake, and weight loss. Reevaluation showed centrilobular ground-glass opacity in both lung fields on chest CT and growth of *Mycobacterium tuberculosis* on sputum acid-fast bacilli culture (Technical Appendix Figure, panel B). A pulmonary tuberculosis infection was diagnosed and treated with antituberculosis medication.

Case-patient 3 (IIBC1303) was a 52-year-old woman hospitalized for fever and left flank pain; her symptoms had persisted for 1 month. She also reported right-side neck swelling and pain at neck levels II, III, and VA. *B. henselae* was isolated from cultures of blood collected on the 16th day of hospitalization. She had no contact with animals.

Case-patient 4 (IIBC1304) was a 42-year-old man we previously reported (*5*) whose main complaints were fever, rash, and arthralgia. *B. henselae* was isolated from a bone marrow sample. The patient had no contact with or experience in raising pets.

Case-patient 5 (IIBC1305), also previously published (*4*), was a 73-year-old woman who had *B. henselae* isolated from her blood. She also did not have any contact with animals.

*Bartonella* species can be grown by blood agar–based culture systems. However, it is difficult to culture them this way because the growth of bacterial cells is slow, and obtaining colonies on the agar plate takes a long time. On the other hand, *Bartonella* species grow more rapidly with cell culture–based systems (*6*). For testing of these patients, we grew ECV304 cells in M199 media containing 10% heat-inactivated fetal bovine serum and inoculated 1 mL of whole blood or other samples from the patients onto the cells. After 24 hours, we washed the cells with Dulbecco’s phosphate-buffered saline and maintained them in M199 media. We performed an immunofluorescence assay (IFA) with the patient’s own serum (1:40 diluted) every week after the inoculation. When the growth of bacteria was observed, we scraped all cultured cells from the T25 flask. We then reinoculated 1 mL of infected ECV304 cells onto uninfected ECV304 cells in a T75 flask for expansion of bacterial cells.

To identify the bacterial isolates, we amplified and sequenced the 16S rRNA gene (*7*). The pathogens cultured from the specimens showed the highest sequence similarities with *B. henselae* Houston-1 strain (GenBank accession nos. KY773227, KY773228, KY773229, KY773290, and KY885188). The similarity was >99% (Figure) (*8*,*9*). IFA results using a commercial *Bartonella* IFA IgG kit (FOCUS Diagnostics, DiaSorin Molecular, Cypress, CA, USA) also showed positive results for all patients’ serum samples; titers ranged from 1:40 to 1:1,280 (Table 1). We also performed multilocus sequence typing to determine the genotypes of *B. henselae* isolates (*10*) and found that all isolates belonged to sequence type 1 (Table 2).

**Figure:**
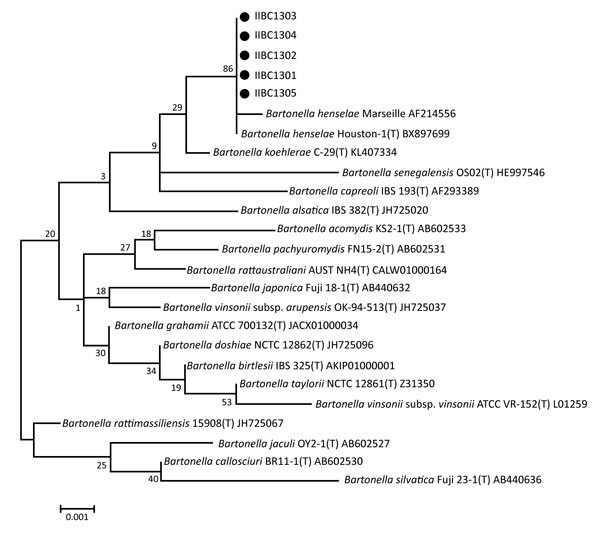
Phylogenetic tree of 5 *Bartonella henselae* clinical isolates from patients in South Korea (black dots) and closely related species based on 16S rRNA gene sequences. Database accession numbers are provided for reference sequences. Scale bar indicates nucleotide substitutions per site.

**Table 2:** Characteristics of clinical *Bartonella henselae* isolates from 5 case-patients, South Korea*

Isolate	Specimen type	Allele at the 8 loci								Sequence type
16S	*batR*	*ftsZ*	*gltA*	*groEL*	*nlpD*	*ribC*	*rpoB*
IIBC1301	Blood	1	1	1	1	1	1	1	1	1
IIBC1302	Blood	1	1	1	1	1	1	1	1	1
IIBC1303	Blood	1	1	1	1	1	1	1	1	1
IIBC1304	Bone marrow	1	1	1	1	1	1	1	1	1
IIBC1305	Blood	1	1	1	1	1	1	1	1	1

## Conclusions

We cultured *B. henselae* isolates from clinical samples and compared characteristics of 5 patients: 3 new cases and 2 previously reported cases from which *B. henselae* was isolated (Table 2). Because of the diverse manifestations of *B. henselae* infection, the symptoms were similar to those of other bacterial infections. *B. henselae* infections in 3 patients were initially misdiagnosed as other diseases: sexually transmitted disease (case-patient 1), enteric fever-like syndrome (case-patient 2), and acute pyelonephritis (case-patient 3). The diagnosis of *B. henselae* infection was made even more difficult because none of these 5 patients reported a history of raising cats. However, the absence of contact with animals should not preclude infection; even though *B. henselae* infection is ususally related to cat scratches or bites, it may also occur without animal contacts (*5*). It is also noteworthy that the patient described in case 2 was co-infected with pulmonary tuberculosis. Co-infection with *B. henselae* and *Mycoplasma* spp. has also been reported in previous studies (*11*,*12*). Co-infection with other bacteria suggests that infection with *Bartonella* species may weaken the host’s immune system, leaving the host vulnerable to secondary infections. In addition, these co-infections may cause difficulty in diagnosing *Bartonella* infection.

Multilocus sequence typing indicated that all isolates from this study belonged to *B. henselae* sequence type 1. This result is consistent with previous studies, which showed relatively less diversity among human strains than among the feline reservoir (*10*,*13*).

In summary, the clinical features of *B. henselae* infection are diverse and nonspecific, which could initially lead to misdiagnosis as other diseases. Physicians and patients should consider that *Bartonella* infection presents various clinical symptoms and might be a common cause of fever of unknown origin, irrespective of exposure to cats. Once *Bartonella* infection is suspected, cell culture should be considered to confirm the diagnosis.

Technical Appendix Clinical imaging of case-patient 2, whose serum sample cultures revealed the presence of *Bartonella henselae*, South Korea.
