# Supplementary material for: Characterization of Clinical Isolates of Bartonella henselae Strains, South Korea
Source: Emerg Infect Dis. 2018 May;24(5):912–5. doi: 10.3201/eid2405.171497 (PMC5938757; doi:10.3201/eid2405.171497)
Supplement: Technical Appendix — Clinical imaging of case-patient 2, whose serum sample cultures revealed the presence of Bartonella henselae, South Korea. [file 17-1497-Techapp-s1.pdf]

Article DOI: https://doi.org/10.3201/eid2405.171497

Characterization of Clinical Isolates of *Bartonella henselae* Strains, South Korea

Technical Appendix

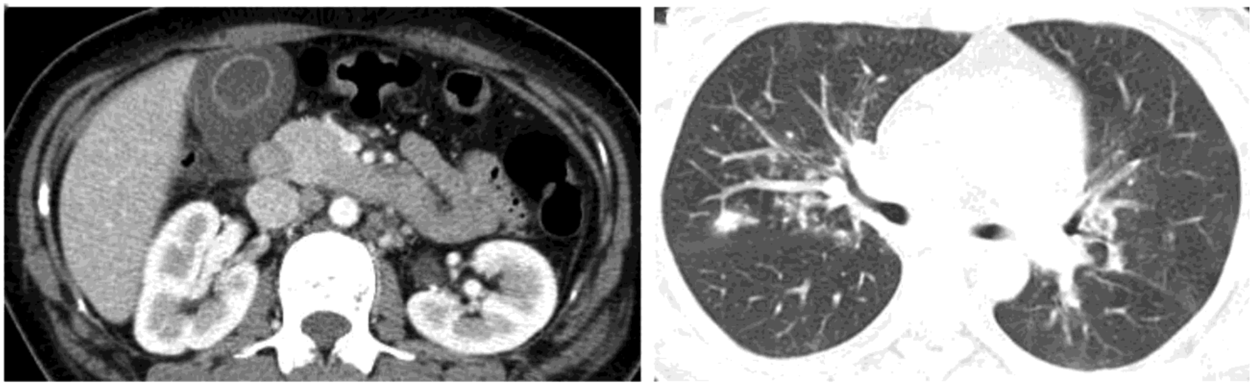

Technical Appendix Figure. Clinical imaging of case-patient 2, a 40-year-old woman whose serum sample cultures revealed the presence of *Bartonella henselae*, South Korea. A) Abdomen–pelvis computed tomography image showing wall thickening of the gallbladder. B) Chest computed tomography image showing centrilobular ground-glass opacity and pneumonia in both lung fields, a finding consistent with pulmonary *Mycobacterium tuberculosis*.
